# The extracellular thioredoxin Etrx3 is required for macrophage infection in *Rhodococcus equi*

**DOI:** 10.1186/s13567-020-00763-3

**Published:** 2020-03-10

**Authors:** Álvaro Mourenza, Cristina Collado, Natalia Bravo-Santano, José A. Gil, Luís M. Mateos, Michal Letek

**Affiliations:** 1 Department of Molecular Biology, Area of Microbiology, University of León, León, Spain; 2 Health Sciences Research Centre, University of Roehampton, London, UK

## Abstract

*Rhodococcus equi* is an intracellular veterinary pathogen that is becoming resistant to current antibiotherapy. Genes involved in preserving redox homeostasis could be promising targets for the development of novel anti-infectives. Here, we studied the role of an extracellular thioredoxin (Etrx3/REQ_13520) in the resistance to phagocytosis. An *etrx3*-null mutant strain was unable to survive within macrophages, whereas the complementation with the *etrx3* gene restored its intracellular survival rate. In addition, the deletion of *etrx3* conferred to *R. equi* a high susceptibility to sodium hypochlorite. Our results suggest that Etrx3 is essential for the resistance of *R. equi* to specific oxidative agents.

## Introduction, methods, and results

*Rhodococcus equi* is an actinobacterial pathogen that can infect immunocompromised humans and foals by causing a fatal pyogranulomatous bronchopneumonia [1]. *R. equi* is distributed worldwide, being highly prevalent in farms because of its colonization of the horse intestine [2]. This pathogen is usually transmitted by inhaling *R. equi*-contaminated dust or respiratory particles produced by infected animals [2].

During the past few decades, a lot of effort has been focused on identifying and studying genes of *R. equi* that could be involved in host–pathogen interactions in search of new strategies to tackle the infections caused by these Actinobacteria. The rise of multidrug-resistant *R. equi* strains is making current antibiotherapies ineffective [3, 4]. In addition, any attempts to develop a vaccine against *R. equi* have been unsuccessful so far [5]. Because of this, hyperimmune plasma administration has been implemented as a preventative primary intervention in foals, despite of its high costs and variable efficacy [6].

It is becoming clear that the virulence associated proteins (Vaps) of *R. equi* are major determinants of the control of intraphagolysosomal pH during cell infection [7]. Furthermore, different members of the pVAP megaplasmids family carry specific complements of *vap* genes, which are essential for the intracellular survival of *R. equi* and they are considered the main driving factor of the host tropism of this pathogen [8].

On the other hand, bacterial proteins involved in redox homeostasis have been traditionally considered very attractive targets for the development of novel anti-infectives against many pathogens [9, 10]. Importantly, *R. equi* is exposed to high concentrations of reactive oxygen and nitrogen species (RONS) during phagocytosis, which may affect membrane lipids, nucleic acids, housekeeping proteins and virulence factors of the pathogen [10]. During phagocytosis, the production of RONS is triggered by the activation of NOX and iNOS proteins in the macrophage [11]. In response, *R. equi* resists the oxidative stress in the phagosome with catalases, superoxide dismutases, alkyl hydroperoxide reductases and thiol peroxidases [12]. Furthermore, *R. equi* is well equipped with protein-repairing genes encoding mycothiol and mycoredoxins (Mrx), which are only present in Actinobacteria [13]. Moreover, there are several genes in the genome of *R. equi* encoding proteins with thioredoxin domains. The main thioredoxin-based antioxidant system is well conserved in bacteria and, in particular, this is encoded by REQ_47340-50 in *R. equi* [12].

However, a detailed analysis of the *R. equi* genome annotation [12] revealed the presence of four genes encoding putative extracellular thioredoxins (Etrx), which were named as Etrx1 (REQ_05180), Etrx2 (REQ_08580), Etrx3 (REQ_13520) and Etrx4 (REQ_37440) (Additional file 1). All four *R. equi* putative Etrx proteins were aligned to extracellular thioredoxins previously studied as important virulence factors in *Streptococcus pneumoniae* [14–16] and *Mycobacterium tuberculosis* [17] (Additional file 1). All Etrx proteins showed a high sequence homology in the domains containing a thioredoxin-active site (WCxxC).

Furthermore, we clustered all Etrx proteins of *R. equi* in an evolutionary distance tree (Additional file 2). Etrx2 of *R. equi* was not rooted with any of the other Etrx proteins included in this analysis, whereas Etrx1 was clustered with CcsX of *M. tuberculosis*. Interestingly, both Etrx3 and Etrx4 were grouped with Rv0526 of *M. tuberculosis*, suggesting that these proteins were two orthologs of Rv0526. Rv0526 has been previously characterized as an extracellular protein anchored to the bacterial membrane in mycobacteria, but very little is known about its possible role in virulence [18].

**Figure 1:**
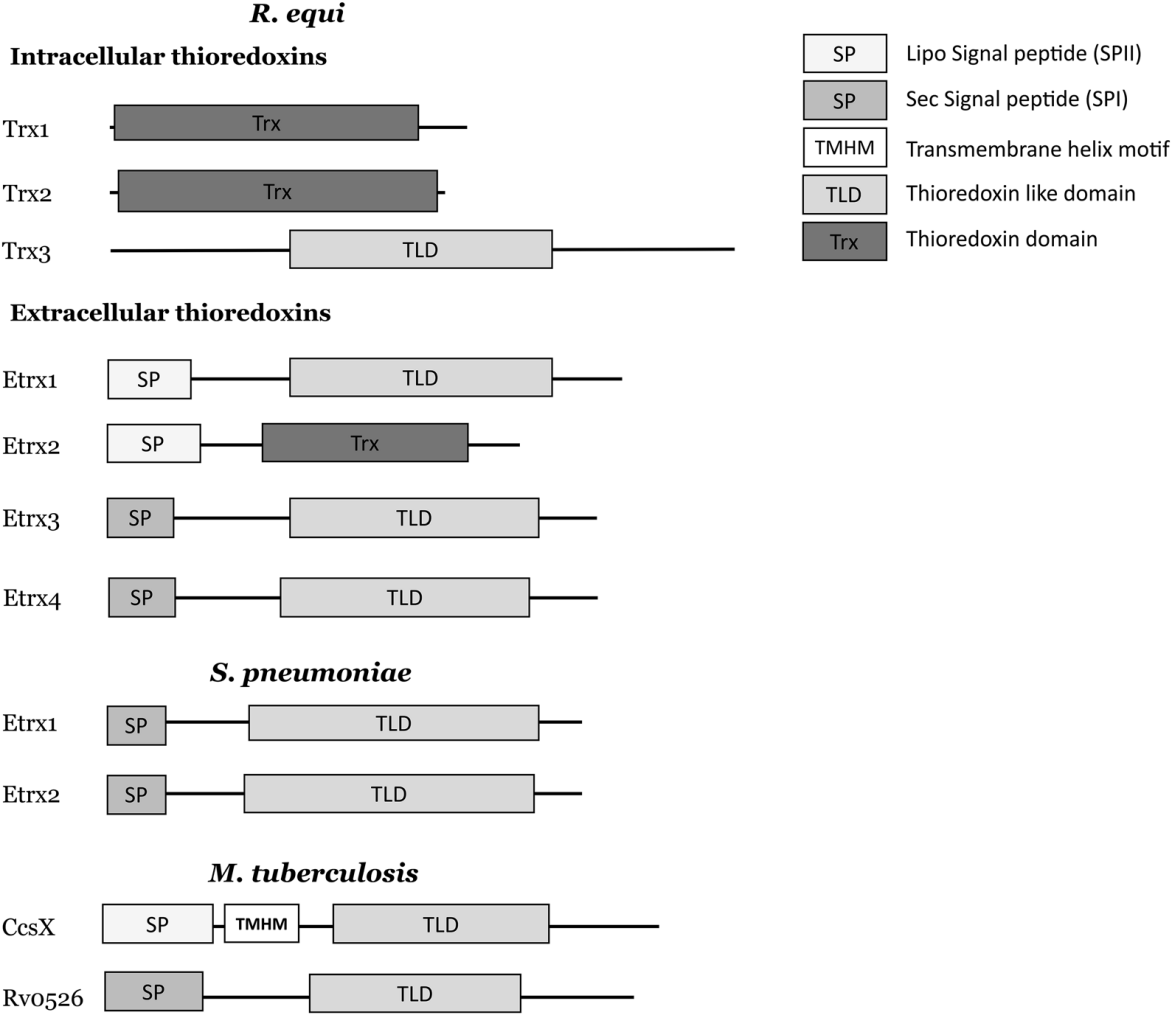
**Protein domains of different extracellular thioredoxins.** The protein sequences of extracellular thioredoxins from *R. equi*, *S. pneumoniae* and *M. tuberculosis* were analysed with SignalP 5.0, TMHMM server, Pfam and pDomTHREADER.

In addition, all Etrx proteins were analysed with SignalP [19], TMHMM [20], Pfam [21] and pDomTHREADER [22] to determine their signal peptides, trans-membrane helix domains and other protein domains (Figure 1). As expected, the overall structure of Etrx3 and Etrx4 was quite similar to that from Rv0526 of *M. tuberculosis* (Figure 1).

Therefore, we analysed the genome regions carrying *etrx*3 and *etrx*4 of *R. equi* and *M. tuberculosis* or the non-pathogenic *Rhodococcus erythropolis* using the Artemis Comparison Tool [23] (Additional file 3). The gene cluster carrying *etrx4* in *R. equi* was inverted but very well conserved in both *M. tuberculosis* and *R. erythropolis* (Additional file 3A). In addition, *etrx3* is an ortholog of Rv0526 of *M. tuberculosis* (Additional file 3B). However, the synteny of the region carrying *etrx3* was very poorly conserved in all three genomes analysed (Additional file 3C). In addition, this region was not acquired by horizontal gene transfer according to a previous analysis of the *R. equi* 103S^+^ genome [12]. This suggested that *etrx3* might have been acquired by a duplication of *etrx4* in *R. equi*, and the new copy of the gene was created in a region made of recurrent genomic rearrangements. Overall, our in silico analysis indicated that Etrx3 might be an extracellular thioredoxin that is unique to *R. equi*. Therefore, we generated an *etrx3*-null mutant strain to study its role in the control of the redox homeostasis of *R. equi* during phagocytosis.

To generate an unmarked in-frame deletion of *etrx3* (REQ_13520) in *R. equi* 103S^+^ (Additional file 4), we amplified by PCR two 1.5 kbp DNA fragments corresponding to the upstream and downstream sequences of the gene (Additional file 5). The resulting amplicons were used as the DNA template of a fusion-PCR reaction to generate a 3 kbp DNA cassette harbouring an in-frame deletion of *etrx3*, which was cloned into pSelAct (Additional file 4) as previously described for the deletion of other genes in *R. equi* [10]. The resulting vector (pSelActΔ*etrx3*—Additional file 4) was electroporated into *R. equi* 103S^+^ and its integration was verified by PCR in several apramycin resistant transformants resulting from the electroporation. The deletion of the *etrx3* gene in *R. equi Δetrx3* was achieved by means of a second recombination event, making use of 5-fluorocytosine counterselection, as previously described [10]. The in-frame deletion of the gene was confirmed by PCR amplification.

To complement the *etrx3*-null mutant with a single copy of *etrx3*, the gene was amplified under the control of its own promoter and cloned in the integrative plasmid pSET152, as described previously for the complementation of other gene deletions in *R. equi* [10]. The resulting vector (pSET*etrx3*—Additional file 4) was used to electroporate *R. equi* Δ*etrx3*, transformants were selected by apramycin-resistance, and the integration of the vector in *R. equi Δetrx3 *+ pSET*etrx3* was confirmed by PCR (Additional file 5). All vectors produced in this study were verified by DNA sequencing.

Optical density at 600 nm (OD_600_) was used to establish the growth curves in trypticase soy broth (TSB) of the mutant strains produced in this study in order to discard any polar effects on their replication rate that could possibly result from genetic engineering. When compared to the wild type strain, the replication rate of both *R. equi* Δ*etrx3* and *R. equi Δetrx3 *+ pSET*etrx3* was unaltered, which facilitated the analysis of their intracellular proliferation rate during infection assays (Additional file 6). Statistical analyses were conducted using IBM^®^ SPSS^®^ statistics v24. One-way ANOVA and post hoc Tukey’s multiple-comparison tests were routinely employed to identify statistically significant differences across conditions in this study.

Macrophage infection assays were performed as previously described [12] using low-passage J774A.1 murine macrophages (American Type Culture Collection) cultured in Dulbecco’s Modified Eagle Medium (DMEM—Thermo-Fisher Scientific). Macrophages were infected at a multiplicity of infection of 10 with exponentially growing cultures (OD_600_ ≈ 1) of *R. equi* in TSB. The presence of the virulence plasmid pVAPA was verified by PCR in all *R. equi* strains tested preceding each infection assay, as previously described [10]. After 1 h of incubation, the medium was replaced with DMEM supplemented with 5 µg/mL vancomycin to kill extracellular bacteria, as previously described [24]. At different time points, cells were lysed with 0.1% Triton X-100 and serial dilutions of the lysates were spread onto trypticase soy agar (TSA) plates for colony forming unit (CFU) counting.

**Figure 2:**
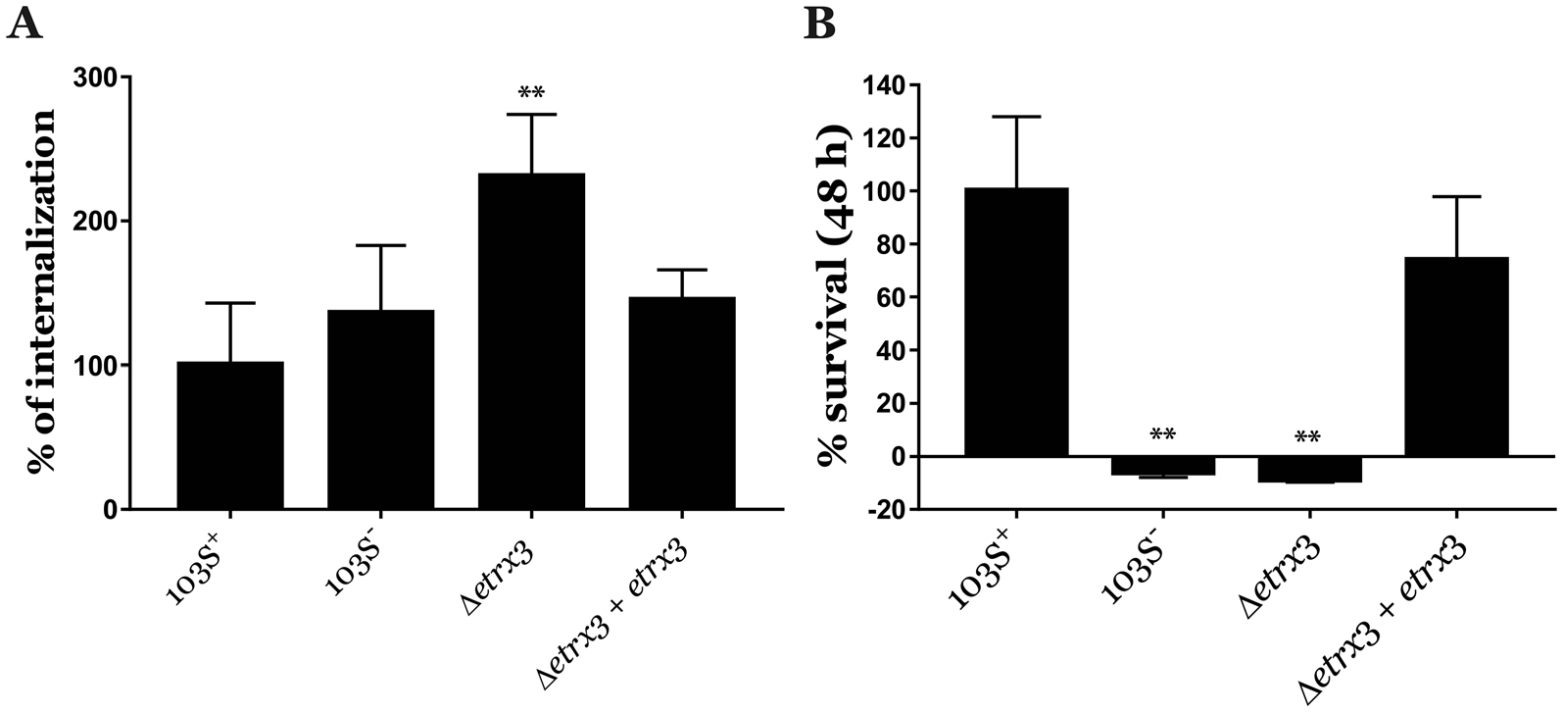
**Macrophage infection assays.** Percentages of internalization (**A**) and intracellular survival at 48 h (**B**) in J774A.1 macrophages of the wild type *R. equi* 103S^+^ strain, the virulence plasmid cured *R. equi* 103S^−^ strain, *R. equi Δetrx*3, and *R. equi Δetrx*3 + pSET-*etrx3* (*Δetrx3* + *etrx3*). Bacterial viability was calculated by quantifying the number of colony forming units (CFUs) of each strain and by normalizing these data against *R. equi* 103S^+^ CFUs. Data are expressed as mean ± SD of three technical replicates repeated in three independent experiments. One-way ANOVA and post hoc Tukey’s multiple comparison tests were performed to assess for statistical significance in relation to the wild type strain. **p-value < 0.01.

We infected J774A.1 cells with *R. equi* Δ*etrx3* and *R. equi* Δ*etrx3* + pSET-*etrx3*. In parallel, we also infected macrophages with the *R. equi* 103S^+^ wild type strain and the virulence plasmid cured derivative *R. equi* 103S^−^, which were respectively considered positive and negative controls of macrophage infection (Figure 2). Interestingly, the internalization rate of the *etrx3*-null mutant strain was significantly higher when compared to the wild type strain (Figure 2A). Despite of this, the *R. equi* Δ*etrx3* strain was unable to persist in the intracellular environment. In contrast, the internalization and intracellular survival of the *R. equi* Δ*etrx*3 + pSET-*etrx*3 complemented strain was comparable to *R. equi* 103S^+^ (Figure 2). Overall, these results suggest that Etrx3 has an essential role in *R. equi*’s macrophage infection.

To cast some light on the role of Etrx3 during phagocytosis, we analysed the resistance of the *R. equi* Δ*etrx3* mutant strain to different oxidative stressors as previously described [10]. Exponential growth phase cultures (OD_600_ = 1) were diluted 1:10 in plain TSB or in TSB supplemented with 10 mM H_2_O_2_, 5 mM NaClO, or minimum medium supplemented with free methionine sulfoxide (MetSO^−^) at different concentrations, and incubated at 30 °C and 220 rpm. At different time points, each culture was serially diluted and spread on TSA plates, which were incubated for 24 h at 30 °C. The number of CFUs was then quantified and results were normalized to the survival rate of *R. equi* 103S^+^. In contrast, to determine the susceptibility to DETA NONOate (a nitric oxide donor), *R. equi* exponential growth phase cultures (OD_600_ = 1) were 1:10 diluted in 10 mL of liquefied TSA (0.6% agar) at 50 °C and spread over 10 mL of settled TSA. Nitrocellulose disks were then placed on the surface of *R. equi*-containing TSA plates. Finally, DETA NONOate was added to paper disks at defined concentrations and plates were incubated at 30 °C for 24 h.

**Figure 3:**
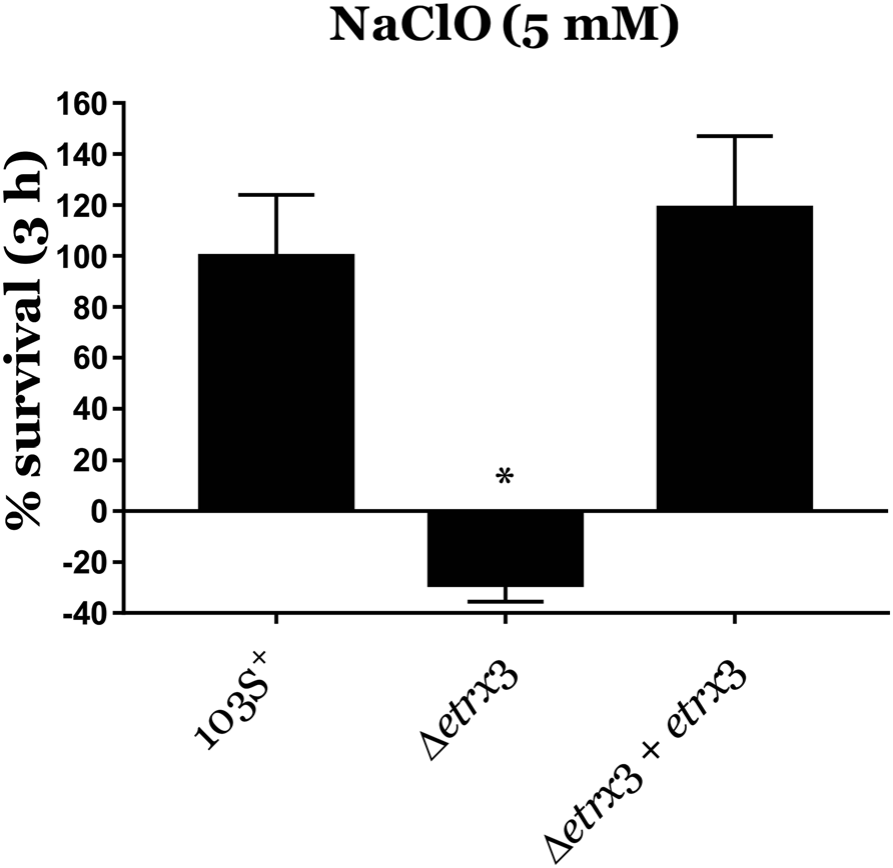
**Resistance of different*R. equi*strains to 5** **mM NaClO.** Data were normalized by the percentage of *R. equi* 103S^+^ CFUs and are expressed as mean ± SD of three technical replicates repeated in three independent experiments. One-way ANOVA and post hoc Tukey’s multiple comparison tests were performed to assess for statistical significance across conditions. *p-value < 0.05.

Our results showed that *R. equi* Δ*etrx3* was significantly more susceptible to sodium hypochlorite than the wild type strain or the *R. equi* Δ*etrx3* + pSET-*etrx3* complemented strain (Figure 3). In contrast, *R. equi*’s resistance to nitric oxide or free methionine sulfoxide (MetSO^−^) was not altered in the *etrx3*-null mutant (Additional files 7 and 8), and its resistance to H_2_O_2_ was even increased (Additional file 9). Importantly, sodium hypochlorite is considered a source of hypochlorous acid, which is produced by a myeloperoxidase expressed in professional phagocytes such as macrophages [25, 26]. Therefore, the low survival rate of *R. equi* Δ*etrx3* within murine macrophages might be due to its high susceptibility to sodium hypochlorite.

**Figure 4:**
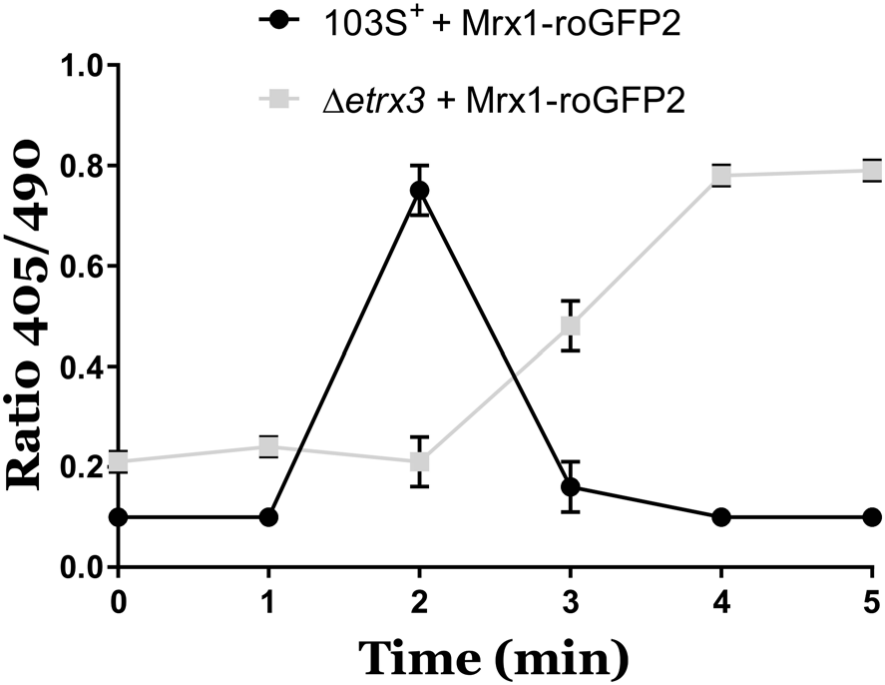
**Ratiometric response of Mrx1-roGFP2 biosensor expressed in*R. equi*103S**^**+**^**and*R. equi*Δ*etrx*3, which were cultured in TSB supplemented with 1** **mM NaClO.** Data represent mean ± SD of three technical replicates repeated in three independent experiments.

To verify this, we also analysed the ratiometric response of Mrx1-roGFP2 in this context, a reengineered redox biosensor that allows to evaluate changes in mycothiol levels in response to an oxidative stressor [10]. The redox status of Mrx1-roGFP2 was measured as described before by means of confocal microscopy [10]. Interestingly, the deletion of *etrx3* in *R. equi* delays the oxidation of Mrx1-roGFP2 caused by NaClO (Figure 4). Overall, our results suggest that Etrx3 has a role in counteracting the redox stress exerted by NaClO.

## Discussion

During phagocytosis, *R. equi* is exposed to RONS generated by host myeloperoxidases, nitric oxide synthases and NADPH oxidases [11]. The control of the pathogen’s extracellular redox homeostasis could be essential to maintain the reduced state and activity of its secreted virulence factors. Otherwise, the reactive oxygen and nitrogen species generated by the macrophage may inactivate essential pathogen’s proteins by oxidation of their cysteine or methionine amino acids.

In Actinobacteria, the thioredoxin/thioredoxin reductase (Trx/TrxR) system act together with the mycoredoxins/mycothiol (Mrx/MSH) system to maintain the reduced state of proteins [13]. The mycoredoxins/mycothiol system restores the reduced state of cysteine residues [13]. The methionine oxidized residues could be reduced by methionine sulfoxide reductases (Msr), which are in turn reduced by a transfer of electrons from the active CxxC site of thioredoxins to the Msr disulphides. Finally, the oxidized thioredoxins are reduced by an NADPH-dependent thioredoxin reductase.

There is an increasing evidence demonstrating the essential role of thioredoxins in the virulence of many bacterial pathogens. In *Listeria monocytogenes*, TrxA maintains the reduced status of the master regulator of virulence PrfA and the key regulator of flagellar synthesis MogR [27]. TrxA is also essential for the intracellular induction of *Salmonella* pathogenicity island 2 (SPI2) type III secretion system (T3SS) and, consequently, for the intracellular replication of *Salmonella enterica* serovar Typhimurium [28].

In addition, several extracellular thioredoxins have been recently described with essential roles in the virulence of *M. tuberculosis*, *S. pneumoniae* or *Agrobacterium tumefaciens* [14–17, 29]. In *S. pneumoniae*, it is becoming clear that the functional paralogues Etrx1 and Etrx2 and the methionine sulfoxide reductase MsrAB2 are part of an extracellular electron pathway. This is required to maintain the redox state of methionine residues present in surface-exposed proteins that are essential for the pathogen’s survival to phagocytosis [14, 15]. However, extracellular thioredoxins may have other functions in the cell. For instance, the extracellular thioredoxin CcsX of *M. tuberculosis* is involved in the maturation of cytochrome *c* oxidase. In all cases, these extracellular redoxins are probably coupled to electron transport chains in the pathogen’s cytoplasmic membrane, which act as the source of their reducing power.

Similarly, here we describe the importance of the extracellular thioredoxin Etrx3 on the intracellular survival of *R. equi*, an actinobacterial pathogen causing infections that are becoming very difficult to treat due to antibacterial resistance. Overall, our data suggest that Etrx3 is essential for the survival of *R. equi* to phagocytosis, and that this extracellular thioredoxin is required to preserve the redox homeostasis of *R. equi* when the pathogen is exposed to NaClO.

However, the high resistance of *R. equi Δetrx3* to H_2_O_2_ suggests that the deletion of *etrx3* leads to a compensatory effect that may implicate the overexpression of other proteins involved in redox homeostasis. Similarly, a *ccsX*-null mutant of *M. tuberculosis* exhibited high resistance to H_2_O_2_ due to the overexpression of the cytochrome *bd* oxidase [17]. In addition, a double *etrx1/etrx2*-null mutant of *S. pneumoniae* was found to be more resistant than the wild type strain to the superoxide-generating compound paraquat [15]. Further studies are necessary to understand the role of Etrx3 in this context. Nonetheless, the high resistance to H_2_O_2_ of *R. equi Δetrx3* had no impact on macrophage infection, since the *etrx3*-null mutant strain was still unable to survive phagocytosis (Figure 2).

On the other hand, the deletion of *etrx3* did not alter *R. equi*’s resistance to the oxidative stress induced by free methionine sulfoxide (Additional file 8). This is in stark contrast to the susceptibility of Etrx1 and Etrx2-null mutants of *S. pneumoniae* to MetSO^−^ [15], suggesting that the role of Etrx3 is not related to the reduction of Msr proteins in *R. equi* (encoded by REQ_01570 and REQ_20650).

Further research is required to elucidate the precise function of Etrx3. However, the essential role of this extracellular thioredoxin during macrophage infection makes the *etrx3*-null mutant strain an attractive candidate for the development of an attenuated vaccine. The *Δetrx3* deletion strain might be able to elicit a strong immune response against *R. equi* since it was unable to survive phagocytosis despite of carrying a functional pVAPA virulence plasmid, which is required to generate both cell-mediated and humoral immune responses [5].

## Supplementary information

**Additional file 1. Sequence alignments of the Etrx proteins of different Gram-positive pathogens.** Re, *Rhodococcus equi*; Mtb, *Mycobacterium tuberculosis*; Sp, *Streptococcus pneumoniae*. Highly conserved regions are highlighted in red. The thioredoxin-active site WCxxC of each Etrx is highlighted with a black rectangle. The alignments were made with T-COFFEE.

**Additional file 2. Unrooted evolutionary distance tree based on amino-acid identity of the putative extracellular thioredoxins from different pathogens.** The tree was constructed by maximum likelihood method using eight Etrx’s. In addition, the *E. coli* Trx1 and *R. equi* Trx conserved cytosolic thioredoxins were included as outgroup. Mt: *Mycobacterium tuberculosis*; Re *Rhodococcus equi*; Sp: *Streptococcus pneumoniae*. The GenBank access numbers are in brackets. Scale represents amino acid changes.

**Additional file 3. Artemis comparison tool (ACT) pairwise chromosome tBLASTx alignment of thioredoxins (in yellow) from different Actinobacteria:*M. tuberculosis*(Rv),*Rhodococcus equi*(REQ),*Rhodococcus erythropolis*(RER).** (A) Overall synteny of the genomic region containing *etrx4*; (B) sequence homology in between *etrx3* and orthologs of *etrx4* in *M. tuberculosis* (Rv0526) and *R. erythropolis* (RER16670); (C) analysis of the genomic region carrying *etrx3* in *R. equi*, *M. tuberculosis* and *R. erythropolis*. Similarity between chromosome regions is depicted by colored lines: in red, sequences in direct orientation; in blue, inverted sequences. Color intensity represents sequence homology percentage, being pink/light blue the lowest and red/deep blue the highest.

**Additional file 4. List of bacterial strains, cell lines and plasmids used in this study.**

**Additional file 5. List of primers used in this study.**

**Additional file 6. Growth curves of*R. equi*103S**^**+**^, ***R. equi*Δ*etrx3*and*R. equi*Δ*etrx3* +** **pSET-*etrx3*strains.** Results were expressed as mean ± SD of three technical replicates repeated in three independent experiments.

**Additional file 7. DETA NONOate susceptibility test.** Analysis of the susceptibility to the oxidative agent DETA NONOate of different *R. equi* strains. Results are expressed as mean ± SD of three technical replicates repeated in three independent experiments. One-way ANOVA and post hoc Tukey’s multiple comparison tests were performed to assess for statistical significance across conditions.

**Additional file 8. Growth curves of*R. equi*strains (*R. equi*103S**^**+**^**and*R. equi*Δ*etrx*3) in minimum medium supplemented with 10 and 30** **mM MetSO**^**−**^. *R. equi* 103S^+^ growing in minimum medium without MetSO^−^ was used as growth control (control). Results were expressed as mean ± SD of three technical replicates repeated in three independent experiments.

**Additional file 9. Percentage of survival of different*R. equi*strains grown in vitro in the presence of 10** **mM of H**_**2**_**O**_**2**_. Data were normalized by the percentage of *R. equi* 103S^+^ CFUs and are expressed as mean ± SD of three technical replicates repeated in three independent experiments. One-way ANOVA and post hoc Tukey’s multiple comparison tests were performed to assess for statistical significance across conditions. (**) *p*-value < 0.01.

## Data Availability

The datasets supporting the conclusions of this article are included within the article and its additional files.

## Abbreviations

CFU: colony forming unit
DMEM: Dulbecco’s Modified Eagle Medium
Etrx: extracellular thioredoxin
Mrx: mycoredoxin
MSH: mycothiol
Msr: methionine sulfoxide reductase
OD_600_: optical density at 600 nm
RONS: reactive oxygen and nitrogen species
Trx: thioredoxin
TrxR: thioredoxin reductase
TSA: trypticase soy agar
TSB: trypticase soy broth
