# Supplementary figures and images for: The extracellular thioredoxin Etrx3 is required for macrophage infection in Rhodococcus equi
Source: Vet Res. 2020 Mar 10;51:38. doi: 10.1186/s13567-020-00763-3 (PMC7063783; doi:10.1186/s13567-020-00763-3)

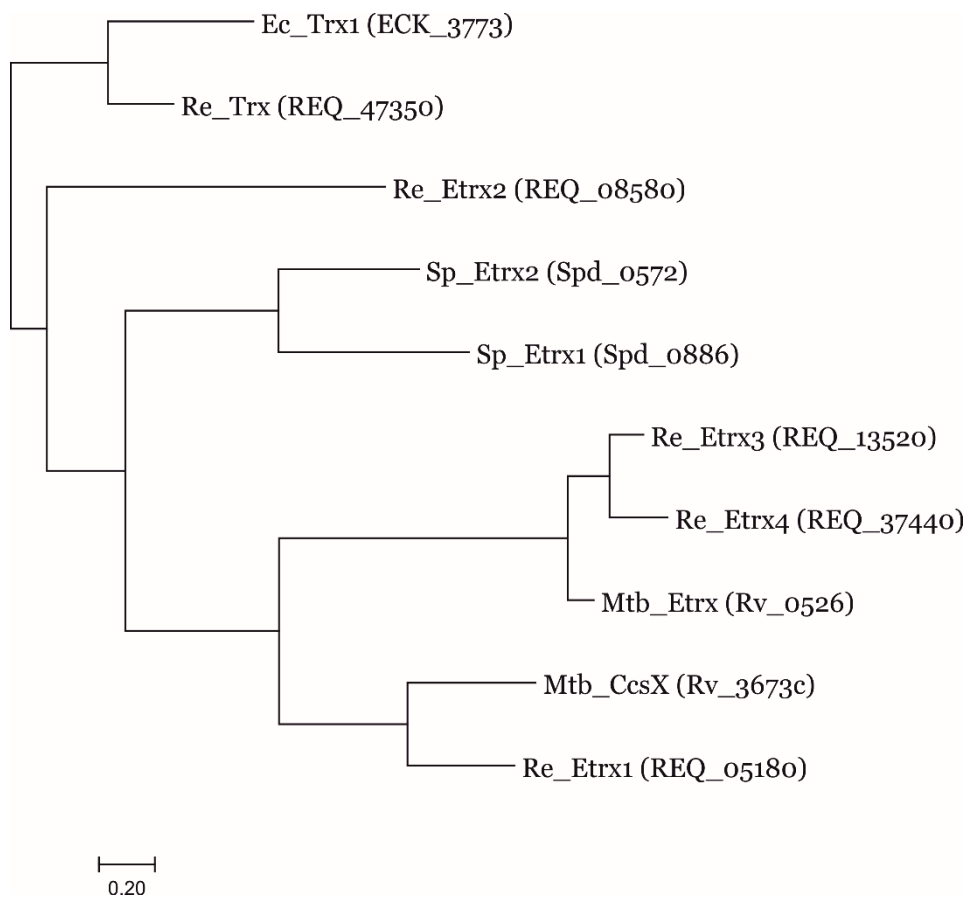

Supplement: Supplementary file 2 — Additional file 2. Unrooted evolutionary distance tree based on amino-acid identity of the putative extracellular thioredoxins from different pathogens. The tree was constructed by maximum likelihood method using eight Etrx’s. In addition, the E. coli Trx1 and R. equi Trx conserved cytosolic thioredoxins were included as outgroup. Mt: Mycobacterium tuberculosis; Re Rhodococcus equi; Sp: Streptococcus pneumoniae. The GenBank access numbers are in brackets. Scale represents amino acid changes. [file 13567_2020_763_MOESM2_ESM.pdf]

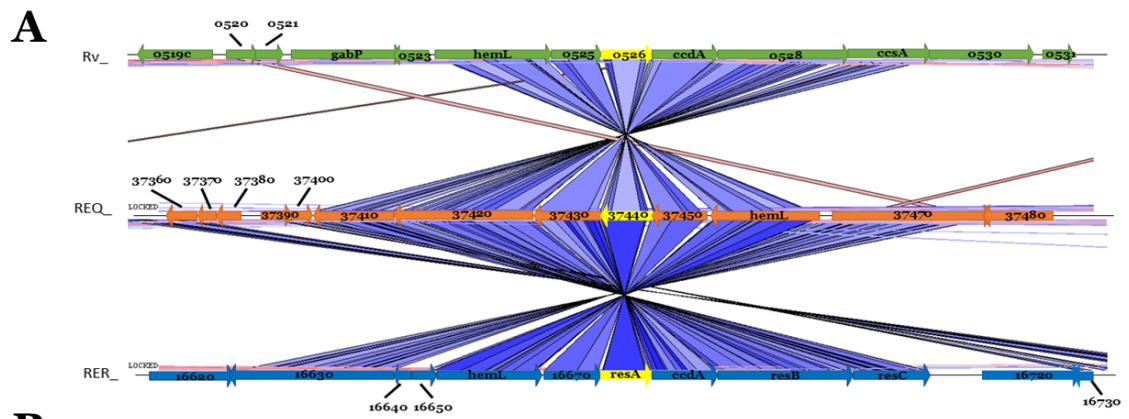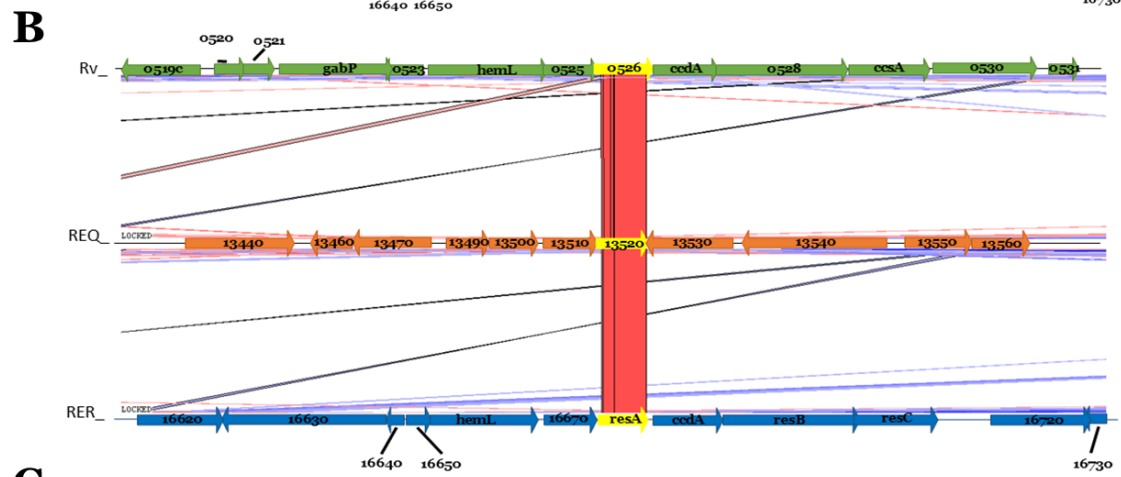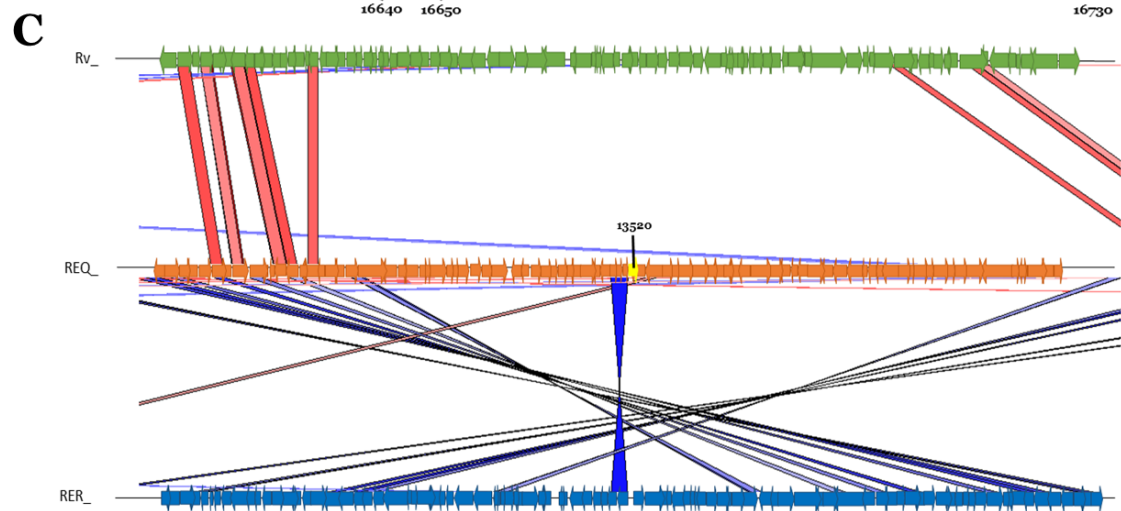

Supplement: Supplementary file 3 — Additional file 3. Artemis comparison tool (ACT) pairwise chromosome tBLASTx alignment of thioredoxins (in yellow) from different Actinobacteria:M. tuberculosis(Rv),Rhodococcus equi(REQ),Rhodococcus erythropolis(RER). (A) Overall synteny of the genomic region containing etrx4; (B) sequence homology in between etrx3 and orthologs of etrx4 in M. tuberculosis (Rv0526) and R. erythropolis (RER16670); (C) analysis of the genomic region carrying etrx3 in R. equi, M. tuberculosis and R. erythropolis. Similarity between chromosome regions is depicted by colored lines: in red, sequences in direct orientation; in blue, inverted sequences. Color intensity represents sequence homology percentage, being pink/light blue the lowest and red/deep blue the highest. [file 13567_2020_763_MOESM3_ESM.pdf]

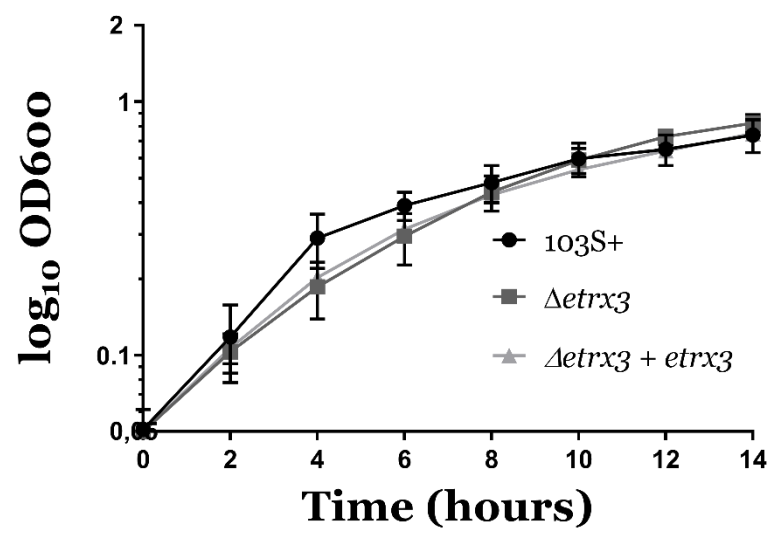

Supplement: Supplementary file 6 — Additional file 6. Growth curves ofR. equi103S+, R. equiΔetrx3andR. equiΔetrx3 + pSET-etrx3strains. Results were expressed as mean ± SD of three technical replicates repeated in three independent experiments. [file 13567_2020_763_MOESM6_ESM.pdf]

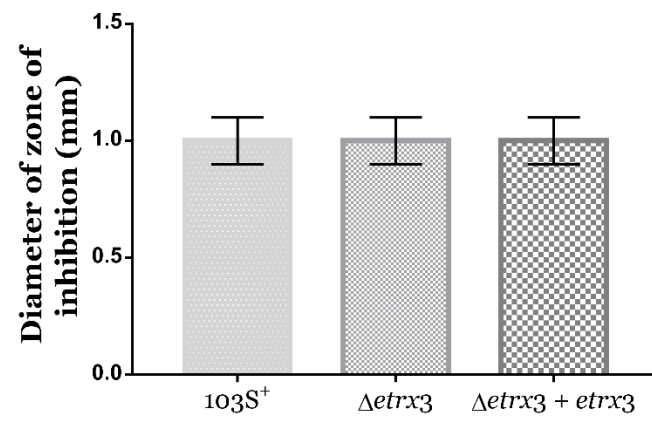

Supplement: Supplementary file 7 — Additional file 7. DETA NONOate susceptibility test. Analysis of the susceptibility to the oxidative agent DETA NONOate of different R. equi strains. Results are expressed as mean ± SD of three technical replicates repeated in three independent experiments. One-way ANOVA and post hoc Tukey’s multiple comparison tests were performed to assess for statistical significance across conditions. [file 13567_2020_763_MOESM7_ESM.pdf]

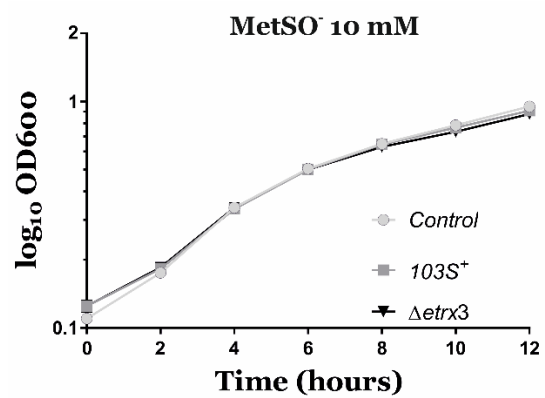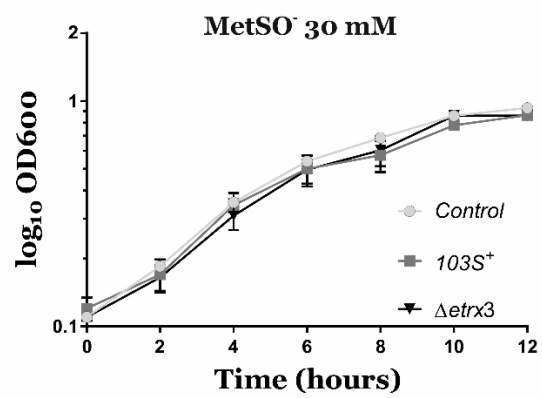

Supplement: Supplementary file 8 — Additional file 8. Growth curves ofR. equistrains (R. equi103S+andR. equiΔetrx3) in minimum medium supplemented with 10 and 30 mM MetSO−. R. equi 103S+ growing in minimum medium without MetSO− was used as growth control (control). Results were expressed as mean ± SD of three technical replicates repeated in three independent experiments. [file 13567_2020_763_MOESM8_ESM.pdf]

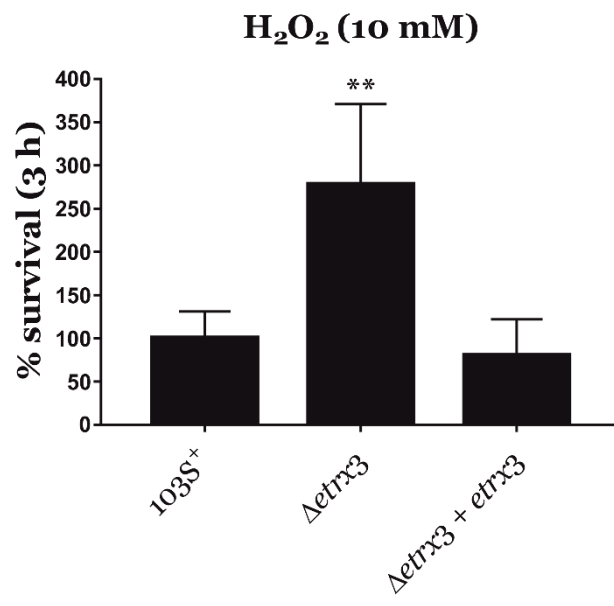

Supplement: Supplementary file 9 — Additional file 9. Percentage of survival of differentR. equistrains grown in vitro in the presence of 10 mM of H2O2. Data were normalized by the percentage of R. equi 103S+ CFUs and are expressed as mean ± SD of three technical replicates repeated in three independent experiments. One-way ANOVA and post hoc Tukey’s multiple comparison tests were performed to assess for statistical significance across conditions. (**) p-value < 0.01. [file 13567_2020_763_MOESM9_ESM.pdf]
