# Supplementary material for: The extracellular thioredoxin Etrx3 is required for macrophage infection in Rhodococcus equi
Source: Vet Res. 2020 Mar 10;51:38. doi: 10.1186/s13567-020-00763-3 (PMC7063783; doi:10.1186/s13567-020-00763-3)
Supplement: Supplementary file 4 — Additional file 4. List of bacterial strains, cell lines and plasmids used in this study. [file 13567_2020_763_MOESM4_ESM.pdf]

Strain or Plasmid	Genotype or characteristics	Source
Escherichia coli DH5α	F- ϕ80d *lacZ*ΔM15 Δ(*lacZYA-argF*) U169 *thi-1 recA1 relA1 endA1 hsdR17* (rk-,mk+) *gyrA96 supE44* λ	Strategene
Rhodococcus equi 103S$^+$	Virulent strain containing pVAPA plasmid	Prof Jesús Navas, Universidad de Cantabria, Spain
Rhodococcus equi 103S$^+$ + Mrx1roGFP2	103S$^+$ derivative, expressing *mrx1-roGFP2*	[10]
Rhodococcus equi 103S$^-$	Plasmid-cured strain of *R. equi* 103S$^+$	Prof Jesús Navas, Universidad de Cantabria, Spain
Rhodococcus equi Δ*etrx3*	*etrx3*-deletion mutant - 103S+ derivative	This study
Rhodococcus equi Δ*etrx3* + pSET*etrx3*	*etrx3* derivative, complemented with *etrx3*	This study
Rhodococcus equi Δ*etrx3* + Mrx1*roGFP2*	Δ*etrx3* derivative, expressing *mrx1-roGFP2*	This study
J774.A1	Mouse BALB/c monocyte macrophages	ATCC
pSET152	φC31 integrase *attP* Apr^R	Dr Mary Hondalus, University of Georgia, USA
pSelAct	Apr^R, *lacZ*, *codA*:*upp*	Prof Lubbert Dijkhuizen, University of Groningen, The Netherlands
pSET*etrx3*	pSET152 containing *etrx3* and 500 bp upstream	This study
pSET*mrx*1*roGFP2*	pSET152 containing *mrx*1 fused to *roGFP2*	[10]
pSelAct*Δetrx3*	pSelAct containing 1500 bp upstream and downstream of *etrx3*	This study
