# Supplementary material for: The extracellular thioredoxin Etrx3 is required for macrophage infection in Rhodococcus equi
Source: Vet Res. 2020 Mar 10;51:38. doi: 10.1186/s13567-020-00763-3 (PMC7063783; doi:10.1186/s13567-020-00763-3)
Supplement: Supplementary file 5 — Additional file 5. List of primers used in this study. [file 13567_2020_763_MOESM5_ESM.pdf]

**Name**	**Sequence**	**Target**
Etrx3 Del F	ATCTAGTCTAGAGGGCGTGTCGTGGCAGATCGTCC	1,500 bp upstream of *etrx3*
Etrx3 Mix R	GCGTCATTCGGCCGCCATCCACACGACACC	1,500 bp upstream of *etrx3*
Etrx3 Mix F	GGTGTCGTGTGGATGGCGGCCGAATGACGC	1,500 bp downstream of *etrx3*
Etrx3 Del R	ATCTAGTCTAGAGCCGGATGGACTCGTACGTCGAA	1,500 bp downstream of *etrx3*
Etrx3 F	ATCTAGCATATGGGGTGTCGTGTGGATGCCG	Complementation of *etrx3*
Etrx3 R	ATTTATCTCGAGGCGTCATTCGGCCGCGATC	Complementation of *etrx3*
VapA F	AGATGAAGACTCTTCACAAGACGG	Amplification of *vapA*
VapA R	CTAGGCGTTGTGCCAGCTACCA	Amplification of *vapA*
Ori pVAP F	TAGCGTATCGATTTAAACAAGCCCGAGCGTCTCGC	Amplification of *ori* pVAPA
Ori pVAP R	GCATCGTTTAAATCGATGCTGCGGGTAACGCAGCTG	Amplification of *ori* pVAPA
