# Supplementary material for: The extracellular thioredoxin Etrx3 is required for macrophage infection in Rhodococcus equi
Source: Vet Res. 2020 Mar 10;51:38. doi: 10.1186/s13567-020-00763-3 (PMC7063783; doi:10.1186/s13567-020-00763-3)
Supplement: Supplementary file 1 — Additional file 1. Sequence alignments of the Etrx proteins of different Gram-positive pathogens. Re, Rhodococcus equi; Mtb, Mycobacterium tuberculosis; Sp, Streptococcus pneumoniae. Highly conserved regions are highlighted in red. The thioredoxin-active site WCxxC of each Etrx is highlighted with a black rectangle. The alignments were made with T-COFFEE. [file 13567_2020_763_MOESM1_ESM.pdf]

```
Re_Etrx1    MRMSSG-------------AR----WS---LVALVV--VVGLIVAIWPRGDDESVDVGG-YPGADRSA---SQQERRAADTPEALAAARA
Re_Etrx2    MTAL---------------------------IILVVAIAATVAVGLI--LOSRAGK---------------------------------V
Re_Etrx3    MWMPG--------------WRN---GR---AIAAAG--LAAMLLSGCATGTDAVAOGGT-FD-FVAPG----GOMDIFYDPPE-------
Re_Etrx4    MKFPS--------------LR----LA---AAACSL--VVAATVAGCATGDDAVAOGGT-FV-FVSPG----GKTELFYD--P-------
Mtb_CcsX    MPSLPTTPAETAMTTLTGKTR----WT---IAILAV--VAALMAALVAQLHDYSAS-ST-I--SORPA----PREHRDGDTPEALAWSRQ
Mtb_Rv0526  MOSRA--------------TRRSGALTMRRLVIAAA--VSALLLTGCS-GRDAVAOGGT-FE-FVSPG----GKTDIFYD--P-------
Sp_Etrx1    MKKWO-------------------------TCVLGA--GSLLC------LTACSGKSV----TSEHOTKDEMKTEOTASKTSAAKGKEVA
Sp_Etrx2    MKKVMF-------------AG----LS---LLSLVV--LMAC-------GEEETKKTQAAQQPKQQT---TVQQISVGKDVP--------

cons        *                                                      .

Re_Etrx1    DAELEPCPAP-GG-PAPDGSVLEGITLECVGDG-SRVDLAA---ALAGKPVLLNLWAYWCGPCREEL----PYLOOYADRA-GSAVTV--
Re_Etrx2    RATAPAATAA-------------------VDDE-RATOLAAAGVTATGRPIVLHFSADWCGPCAAVRRVVGOVLARFEADP-DGTPAATE
Re_Etrx3    --------S-RGTIGN-----LAG--PELI-TD-OOVGVA----DFPGOVVVINLWGOWCAPCRAEA----DDLERVYOATRGSGVOF--
Re_Etrx4    -------PSERGAVGK-----VSG--EDLMTDG-KTTSLT----DYEGOVVVLNVWGOWCGPCRGEA----DDLEKVYENTKDMGVOF--
Mtb_CcsX    RANLPPCPAA--G-NGPGAAALRGVVVVCAGDG-SAVDVAR---ALAGRRVVINLWAHWCAPCMTEL----PVMAEYORRV-GPAVLV--
Mtb_Rv0526  -------PASRGR-PGP----LSG--PELA-DPARSVSLD----DFPGOVVVVNVWGOWCGPCRAEV----SOLORVYDATRGAGVSF--
Sp_Etrx1    D--------------------FE---L-MGVDG-KTYRLS----DYKGKKVYLKFWASWCSICLASL----PDTDEIAKEA-GDDYVV--
Sp_Etrx2    D--------------------FT---L-QSMDG-KEVKLS----DFKGKKVYLKFWASWCGPCKKSM----PELMELAAKP-DRDFEI--

cons                                      :        *: : ::. . **. *            .      .

Re_Etrx1    LTVHT-----DPNEANALARLAEYDV-HLPGVQDGSARVQAAVG---SPAVLPV-SVL-------IRPDGTVAKILPQPFRSVDE--IAD
Re_Etrx2    VEVDI-----D--EHPALAK---------------------T-L---GVLSLPTTFILDGALTERFRISGVPSA---------PD--LET
Re_Etrx3    LGINVR----DPORDKAODFAVDFGL-TYPSIYDPPMRSLLALGGKYPTSVIPS-TIV-------LDROHRVAAVFLRELLDVDLQPVVE
Re_Etrx4    LGINVR----DHTKDKAODFVVNNKV-GYPSIYDPEMRTLLTLGKNYPTSVIPT-TIV-------LDREHRVAAVFLKELLVED---LOP
Mtb_CcsX    VTVHO-----GONEAAALSRLADLGV-RLPTLODDRRRVAAALR---VANVMPA-TVV-------LRPDGSVAOTLPRAFGSADE--IVA
Mtb_Rv0526  LGIDVR----DNNROAPODFINDRHV-TYPSIYDPAMRTLIAFGGKYPTSVIPS-TLV-------LDROHRVAAVFLRELLAADLQPVVE
Sp_Etrx1    LTVVSPGHKGEOSEADFKNWYKGLDYKNLPVLVDPSGKLLETY----GVRSYPT-OAF-------IDKEGKLVKTHPGFM-EKDA--ILO
Sp_Etrx2    LTVIAPGIQGEKTVEQFPQWFQEQGYKDIPVLYDTKATTFQAY----QIRSIPT-EYL-------IDSQGKIGKIQFGAISNADA---EA

cons        : :                                      :          *    .       :  .

Re_Etrx1    VVO------OYL---G--V--AA--
Re_Etrx2    ALA------PLLETGR--P--NS--
Re_Etrx3    ------RIAA--------------E
Re_Etrx4    VVERIAQEPODO---G---------
Mtb_CcsX    AVG------NDA----------G--
Mtb_Rv0526  ------RVAEEE---PSGRAPVGAQ
Sp_Etrx1    TLK------ELS-------------
Sp_Etrx2    AFK------EMN-------------

cons
```
